# Approaches, enablers and barriers to govern the private sector in health in low- and middle-income countries: a scoping review

**DOI:** 10.1136/bmjgh-2024-015771

**Published:** 2024-11-13

**Authors:** Catherine Goodman, Sophie Witter, Mark Hellowell, Louise Allen, Shuchi Srinivasan, Swapna Nixon, Ayesha Burney, Debrupa Bhattacharjee, Anna Cocozza, Gabrielle Appleford, Aya Thabet, David Clarke

**Affiliations:** 1 London School of Hygiene & Tropical Medicine, London, UK; 2 Institute for Global Health and Development and ReBUILD for Resilience Consortium, Queen Margaret University, Edinburgh, UK; 3 University of Edinburgh School of Social and Political Science, Edinburgh, UK; 4 Oxford Policy Management, Johannesburg, South Africa; 5 Oxford Policy Management, New Delhi, Delhi, India; 6 Oxford Policy Management, Islamabad, Pakistan; 7 Special Programme on Primary Health Care, World Health Organization, Geneva, Switzerland

**Keywords:** Global health, Health policies and all other topics, Health policy, Health systems, Public health

## Abstract

**Introduction:**

The private sector plays a substantial role in delivering and financing healthcare in low- and middle-income countries (LMICs). Supporting governments to govern the private sector effectively, and so improve outcomes across the health system, requires an understanding of the evidence base on private health sector governance. This paper reports on a scoping review, which synthesised evidence on the approaches used to govern private sector delivery and financing of healthcare in LMICs, the effectiveness of these approaches and the key enablers and barriers to strengthening governance.

**Methods:**

We undertook a systematic search of databases of published articles and grey literature to identify eligible papers published since 2010, drawing on WHO’s governance definition. Data were extracted into a pretested matrix and analysed using narrative synthesis, structured by WHO’s six governance behaviours and an additional cross-cutting theme on capacities.

**Results:**

107 studies were selected as relevant, covering 101 LMICs. Qualitative methods and document/literature review were predominant. The findings demonstrate the relevance of the WHO governance behaviours, but the lack of robust evidence for approaches to implementing them. Valuable insights from the literature include the need for a clear vision around governance aims; the importance of ensuring that policy dialogue processes are inclusive and transparent, avoiding interest group capture; the benefits of exploiting synergies between governance mechanisms; and the need to develop capacity to enact governance among both public and private actors.

**Conclusion:**

Governance choices shape not just the current health system, but also its future development. Common barriers to effective governance must be addressed in policy design, stakeholder engagement, public and private sector accountability, monitoring and capacity. Achieving this will require in-depth explorations of governance mechanisms and more rigorous documentation of implementation and outcomes in diverse contexts.

**WHAT IS ALREADY KNOWN ON THIS TOPIC**

- The private sector plays a substantial and expanding role in delivering and financing healthcare in most low- and middle-income countries (LMICs).
- The private healthcare sector in many LMICs is argued to be undergoverned, detrimentally impacting on patients’ rights and safety and national health policy goals.
- There is increasing interest in the governance of private healthcare, and a growing literature in this area, covering experiences across diverse settings.

**WHAT THIS STUDY ADDS**

- This is the first comprehensive review of governance of the private healthcare sector in LMICs.
- It describes the approaches used for private sector governance, structured by WHO’s six governance behaviours, and synthesises the evidence on their effectiveness and enablers/barriers.
- The review emphasises the importance of private sector governance in shaping the current health system and its future development.

**HOW THIS STUDY MIGHT AFFECT RESEARCH, PRACTICE OR POLICY**

- Insights include the need for a clear vision for the private sector’s role, inclusive and transparent policy processes, effective management of conflicts of interest and development of key capacities and institutions in public and private sectors.
- More robust empirical studies are needed on governance interventions, with particular gaps including private health insurance regulation, public accountability mechanisms, digital health, medical tourism and governance strategies in fragile and conflict-affected settings.
- The findings have informed the development of a ‘Progression Pathway for the Governance of Mixed Health Systems’ to support countries in strengthening governance.

## Introduction

 The private sector plays a substantial role in delivering and financing healthcare in most low- and middle-income countries (LMICs).^1 2^ Defined as all individuals and organisations involved in provision of health services that are neither owned nor directly controlled by government,^3^ the private sector encompasses both for-profit and not-for-profit entities, including private hospitals and clinics, laboratories, blood banks, pharmacy retailers and wholesalers, equipment providers, private insurers and health maintenance organisations (HMO). The role and scope of private sector involvement in healthcare has expanded rapidly in recent decades, driven by income growth, urbanisation and dissatisfaction with the coverage and quality of public provision.^1 4^ Private providers play a substantial role in the delivery of both ambulatory and inpatient care, including for maternal and child health services, and infectious and non-communicable diseases, with different segments of the private sector serving different income groups.^5^,^7^

Historically, LMIC governments perceived their main role to be the finance and delivery of healthcare through public facilities, with engagement with the private sector restricted to fairly minimal regulation of private providers and, in some contexts, partnerships with faith-based organisation (FBO) facilities. In recent years, greater interest has emerged in the government’s role in governing private healthcare more broadly,^8^ reflecting both the growth in this sector, and recognition that achieving universal health coverage (UHC) in a context of ageing populations, evolving disease burden and fiscal pressures will require states to harness the infrastructure, resources and skills of both the public and private sectors.^9^ However, currently the private sector is arguably undergoverned in many LMIC settings, with the government’s efforts to incentivise and control private behaviour seen as inadequate in terms of safeguarding patients and achieving health policy goals.^4^

Multiple definitions of health system governance have been proposed.^10^ It is defined by the WHO as ‘ensuring strategic policy frameworks exist and are combined with effective oversight, coalition-building, regulation, attention to system design and accountability’.^11^ WHO’s own focus on the private sector has evolved over the past 25 years. In 2010, the World Health Assembly called for enhanced engagement, oversight and regulation of private healthcare providers.^12^ In 2019, a WHO call to action emphasised the importance of private sector engagement in advancing the Sustainable Development Goals agenda.^9^ In response, WHO established a Technical Advisory Group on the Governance of the Private Sector for UHC, which developed a strategy entitled ‘Engaging the private health service delivery sector through governance in mixed health systems’.^13^ This strategy drew on work by Travis *et al*^14^ on health system stewardship functions to articulate six governance behaviours required for the effective governance of mixed health systems (table 1).^15^

**Table 1:** The six governance behaviours^15^

Governance behaviour	Definition
Deliver strategy	Government articulates clear strategic objectives for the health system and the role of the private sector.
Enable stakeholders	Government influences private sector operation and performance through financing and regulatory policies.
Foster relations	Inclusive policy processes involve a broad range of stakeholders, including the private sector.
Build understanding	Government ensures access to comprehensive and high-quality data on the private sector’s operation and performance.
Align structures	Actions are taken to coordinate and align private and public sectors in service delivery.
Nurture trust	Measures are implemented to safeguard patient rights and financial welfare in private sector interactions.

Supporting LMIC governments to effectively implement these behaviours requires a detailed understanding of the evidence base on private healthcare governance in these settings. While existing literature reviews cover various aspects of health system governance more broadly, or in specific settings, for specific purposes or through specific mechanisms,^1016^,^32^ a comprehensive review on the governance of the private health sector is lacking. A scoping review was therefore commissioned by WHO to synthesise the existing literature on the six governance behaviours in LMICs. The review explored three research questions for each of the governance behaviours:

- How do various entities approach the governance of the private health sector in LMICs?
- To what extent are these approaches effective in governing the private health sector?
- What are the key enablers and barriers to the adoption of governance approaches, and what potential avenues have been identified to strengthen governance?

Given the exploratory nature of the review, the broad scope of the research questions, and their application to an extremely diverse set of governance approaches, using a wide range of qualitative and quantitative evidence types, it was not appropriate to apply systematic review methods.^33^ We therefore used a scoping review methodology to both summarise the nature and coverage of the literature and provide a descriptive synthesis of the findings.^33 34^

## Methods

### Eligibility criteria, screening and article selection

We adopted WHO’s definition of health system governance provided above of ‘ensuring strategic policy frameworks exist and are combined with effective oversight, coalition-building, regulation, attention to system design and accountability’^11^ and further drew on the conceptualisation of the governance behaviours^13 15^ to identify eligible papers. We included papers concerning governance of any for-profit and not-for-profit, formal and informal private actors having a role in health financing and service delivery in any low- or middle-income country, but excluded other private actors such as manufacturers, social care, training institutions and producers of unhealthy commodities. We included papers concerning governance at national or subnational level but excluded papers on global/multilateral governance. We took a broad approach to eligibility by publication type, including published and grey literature, and quantitative and qualitative methods, literature reviews and evidence syntheses, in all languages (we did not include original policy, regulatory or legislative documents). We restricted the search to papers published from January 2010 to ensure relevance of the health systems context to the present day (while allowing rare exceptions for earlier seminal pieces). Full details on the inclusion criteria are provided in online supplemental table S1.

Systematic searches were conducted in Medline OVID, Scopus and Web of Science in January 2023 using free text and MeSH terms related to the domains of ‘private sector’ and ‘governance’ and ‘health’ (for full search strategies see online supplemental materials 2). Grey literature was identified through expert consultations and by searching the publication repositories of a range of websites of large international non-governmental organisations (INGOs), donor bodies, grant organisations and universities (eg, Results for Development, WHO, World Bank e-Library, Institute of Development Studies, University of Sydney, Lee Kuan Yew School of Public Policy) using a shorter set of search terms. Three reviewers (SS, SN, AB) piloted the screening process on a common set of articles, and then each screened a tranche of the remaining articles independently, with support from all other authors to discuss articles for which inclusion was uncertain, with final decisions reached through consensus.

Given the very large number of items identified through database searches, title and abstract screening was conducted using the ASReview machine learning tool to prioritise papers for screening. In line with previous practice, the reviewers screened at least 10% of their individual tranches and then continued to screen until they had identified 50 irrelevant articles in a row (the stopping rule).^35^,^37^ Full-text review was conducted for all articles screened as potentially eligible and of grey literature.

### Data extraction and analysis

Data extraction was performed using a pretested matrix in Microsoft Excel independently by four reviewers (SS, SN, AB, DB), with support from all authors. Data extraction focused on the three research questions. We extracted information from the literature reviews using the same approach that we used for empirical papers; we did not go back to the original sources or try to distinguish between evidence synthesised from pre-2010 and post-2010 papers. A narrative synthesis was conducted, with findings organised around the three research questions for each of the six governance behaviours, and an additional cross-cutting theme on capacities for governance.

### Patient and public involvement

No patients or members of the public were involved in this study.

## Results

### Summary of the literature included

**Figure 1:**
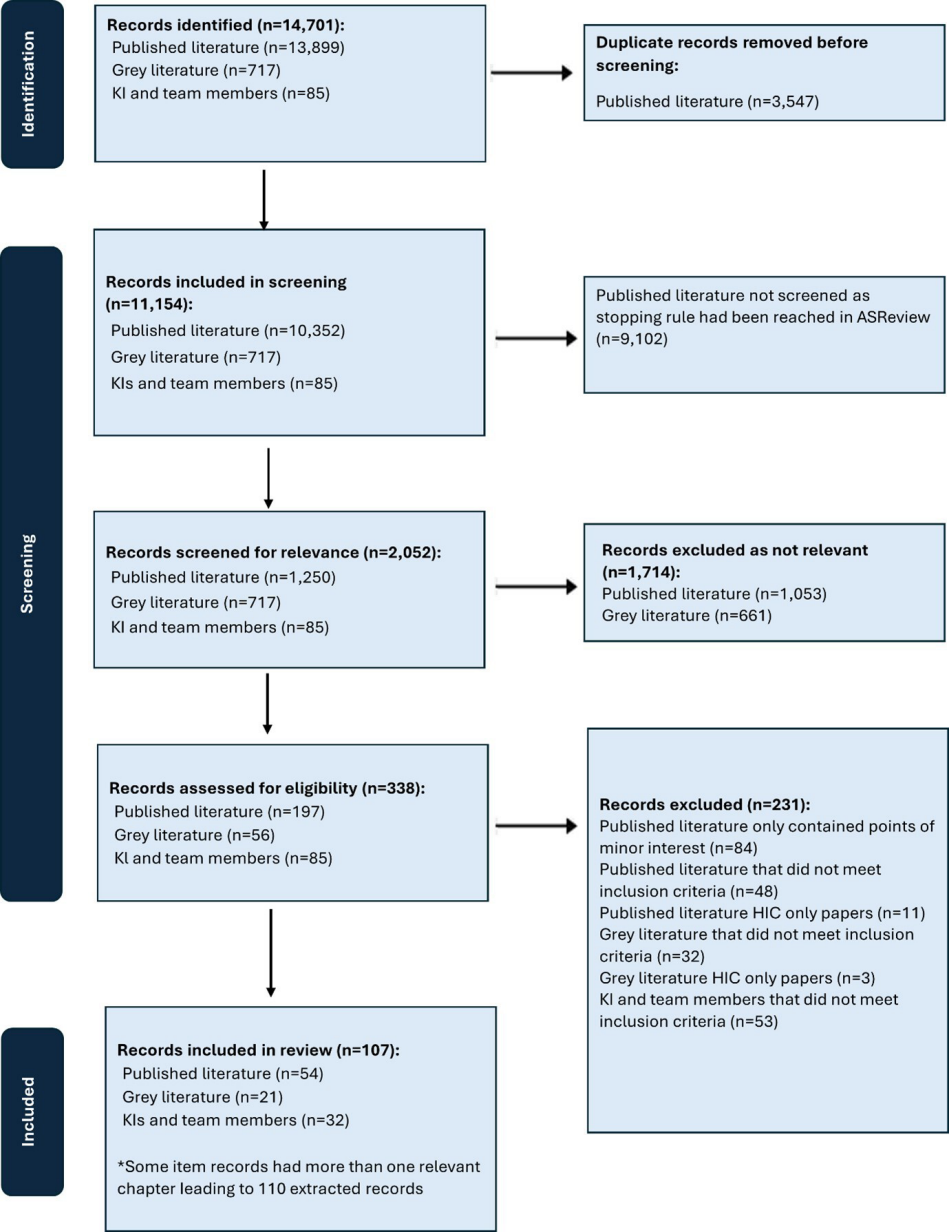
Preferred Reporting Items for Systematic Reviews and Meta-Analyses (PRISMA) flow diagram. KI, Key informants; HIC, High income country.

Our initial searches identified 13 899 records from databases, 717 reports identified through the web search and 85 papers from experts before deduplication. Following deduplication and screening, 338 records were selected for full-text review, of which 107 were selected for inclusion, or 110 items (some documents were books with more than one relevant chapter) (figure 1). We included three papers published pre-2010 that were considered highly significant in the development of the study of private sector governance.^38^,^40^

The characteristics of the included papers are shown in table 2 (more detail on each individual study is provided in online supplemental materials 1). The largest category of private actors studied was private facilities, followed by private health insurers and non-governmental organisations (NGOs). Regulation and contracting were the most common governance mechanisms discussed. Papers were identified covering 101 LMICs, including some from all six WHO regions. We found the highest concentration of papers covering LMICs in the African Region, followed by the South-East Asian Region and Eastern Mediterranean Region. Concerning the research methods used, qualitative methods and document/literature review were predominant.

**Table 2:** Characteristics of included studies

Paper characteristics*	Total papers extracted: 110
Number of papers	110
Types of private sector actors covered	
Private healthcare facilities (hospitals, health centres, clinics, etc)	92
Private insurance companies or HMOs	30
Non-governmental organisations (NGOs) (national and international)/civil society organisations (CSOs)	24
Pharmacies and other retailers	21
Laboratories	7
Governance tools discussed	
Regulation/legislation	62
Contracting/purchasing	47
Support/collaboration/guidance	22
Accreditation	8
Taxation	1
Public accountability mechanisms	5
Level of governance covered	
National	88
Subnational	30
WHO regions of LMICs covered	
African Region (AFR)	63
Region of the Americas (AMR)	24
South-East Asian Region (SEAR)	43
European Region (EUR)	16
Eastern Mediterranean Region (EMR)	29
Western Pacific Region (WPR)	27
Journal/publication type	
Health and health systems journals	62
Social policy and development journals	11
Reports	27
Other†	9
Data collection methods	
Qualitative study	45
Quantitative study	3
Mixed methods	19
Reviews (document/literature)	38
N/A‡	5

* Many publications include more than one category of private sector actor, governance tool, WHO region or data collection method.

† Other publications include book chapters, policy briefs and academic theses.

‡ These are papers which do not employ any data collection method and which are conceptual in nature.

HMOs, health maintenance organisations; LMICs, low- and middle-income countries.

We present the findings, organised by governance behaviour.

### Deliver strategy

Deliver strategy is about articulating clear strategic goals and objectives for the health system and a clear definition of roles for the private health sector in achieving these. To assess whether the government had defined clear roles for the private sector, we considered evidence on the existence of up-to-date policies (eg, legal documents or policy statements) that define clear objectives for the private sector, in line with health system goals, and an articulation of how specific policy mechanisms will be used to influence the operation and performance of the private sector in line with these objectives.^15^

The literature indicates widespread inclusion of the private sector in national health sector strategies, policies and plans. A 2011 assessment found that over 85% of African countries mentioned the private sector in national policies, though the content varied from just recognising its role to giving it a prominent position in achieving strategic aims.^41^ A more recent 2020 assessment of 17 LMICs reported that all countries included the private sector in policies or strategic plans, though only a few had established a formal partnership framework.^2^ Specific private health sector or public–private partnership policies were observed in a minority of LMICs,^241^,^44^ and in some settings vertical programmes had their own private sector strategies.^28 45^,^47^

There is consensus across the literature on the importance of strong strategic policy direction for private sector governance.^241 45 48^,^51^ However, there is limited evidence on the specific impact of including the private sector in policy on subsequent governance arrangements, perhaps reflecting that it is challenging to separate this impact from other drivers for enhanced governance. There is evidence that in many settings inclusion in policy documents has not translated into a clear vision for the private sector’s role, and that implementation is limited.^2 8 41 46 51 52^

In earlier years, a barrier to the inclusion of the private sector in policy was that its governance was not seen as a priority for Ministries of Health (MoHs). Other barriers included a lack of relevant skills and funding in the public sector, an absence of private provider organisations to interface with and mutual mistrust.^43 53 54^ In some settings, such as India, this was reinforced by resistance from private actors to greater regulation.^54^ More recently, there has been increased focus on private sector inclusion in health policy, facilitated by better information on its growing size and complexity.^46^ Other factors stimulating greater inclusion of the private sector in policy have included the influence of external donors and technical advisors, and perceived positive results from private sector engagement in priority health programmes such as family planning and tuberculosis (TB).^43 44 46 51 54^

### Enable stakeholders

Enable stakeholders covers the government’s use of regulatory and financing policy mechanisms to influence the private sector, with the principal financing mechanism being purchasing/contracting. We summarise the literature on these two broad areas in turn.

#### Regulation

Regulation of the private health sector spans diverse legal frameworks, covering public health, professional standards, legal redress, oversight of facilities, pharmacies and private insurers, and broader economic regulations.^42 43 48 55 56^ Core components include licensing facilities and practitioners, setting quality and safety standards, and compliance monitoring.^57^,^59^ While most regulation focuses on quality and safety standards, occasionally it extends to geographical location, for example, requiring Certificates of Need for new facilities,^50^ or stipulating minimum distances between pharmacies.^4 60^ Other requirements may include mandatory reporting of data, ensuring access to emergency services and restricting advertising.^38 61 62^ Some countries regulate prices,^2 22 54 57 58^ with concern about profiteering during the COVID-19 pandemic leading to additional price caps in some countries.^63 64^ Voluntary or mandatory facility accreditation also forms a key component of the regulatory system in some settings.^8 20 54 65^ Registration of healthcare insurers generally focuses on financial soundness, though some countries also regulate enrolment, benefits and risk rating.^22^

There is considerable evidence of poor regulatory compliance across multiple LMICs and provider types. Studies, particularly from the earlier period of the review, highlighted high numbers of unlicensed providers in many settings,^38 41 66^ though some countries have made substantial progress in this area.^52 61^ Other widespread forms of non-compliance included poor adherence to minimum standards, clinicians or pharmacists operating more establishments than legally allowed, public sector staff also working in the private sector even where such dual practice was outlawed, provision of unnecessary services, and breaches in pharmacy practices, notably provision of prescription-only medicines without a prescription.^20 22 27 48 50 56 60 67 68^

There is limited rigorous evidence on interventions to enhance regulation.^24 25 27^ An exception was a randomised controlled trial (RCT) of facility regulation reforms in Kenya, which led to a substantial increase in inspection scores,^68^ at an annualised economic cost of US$311 per inspection.^69^ A Cochrane review also reported positive effects from RCTs of enhanced pharmacy regulation in Thailand, Vietnam and Laos but these were published in the early 2000s, before our review period.^25^ Turning to voluntary accreditation or certification, an RCT of private sector certification in Tanzania found a small increase in compliance with certification standards, but no improvement in clinical quality.^70^ A Cochrane review reported similar results from one 2003 South African study, again outside our review period.^24^ A review with less restrictive inclusion criteria found multiple studies showing positive effects of accreditation on quality, though a minority found no impact.^20^

The few papers addressing the effectiveness of health insurance regulation emphasised its importance for the development of equitable, efficient and high-quality health systems.^71^,^74^ In South Africa, 1990s deregulation was said to have led to ‘dramatic unintended consequences’ in terms of private sector growth, increased costs and exclusion of high-risk patients.^22 73^ In Thailand, progress towards UHC was argued to be facilitated by strict underwriting requirements that constrained insurance market development; in contrast, in Brazil, limited regulation, and tax breaks for insurers, empowered private actors, negatively affecting UHC progress,^71^ and leading to poor-quality and inefficient provision.^75^ Similar themes were raised in the literature on medical tourism, with concerns that regulatory neglect could distort health systems by attracting scarce personnel from the public sector, prioritising tertiary services and worsening inequalities.^71 76 77^

Multiple barriers to effective regulation were raised in the literature. First, the complex legal environment, compounded by rapidly changing health technologies, leads to gaps, overlaps and inconsistencies between laws.^43 48 50 56 78^ Duplicative mechanisms included requirements to register with multiple bodies,^22 65^ while gaps include the ‘informal’ sector, often ignored by regulators because it is considered illegal.^56 79^

Second, there is considerable evidence, particularly in lower income settings, that regulation is poorly implemented: there is limited follow-up post registration, with inspections rare or sporadic, and sanctions often not applied.^2 4 22 41 48 50 54 56 57 66 68 78 80 81^ Implementation is complicated by overlapping mandates of multiple bodies, inadequate incentives for inspectors, their imperfect access to information and under-resourcing.^2 4 22 41 56 66 82 83^

For regulation to be effective, private actors must be able to comply within their economic constraints.^4 57^ High licensing and accreditation fees can be a barrier to compliance.^4 38 43 56 65 82^ Moreover, some providers argue that requirements to locate in rural areas, adhere to price caps or refrain from selling prescription medicines are financially unviable,^52 60 64 84^ with regulators sometimes accepting that enforcing standards could reduce access for poor communities.^4^ The resulting divergence between regulations and common practice provides extensive opportunities for corruption.^4 50 56 60 81 82 85^

Several authors stress that regulation is inherently a political process, with vested interests influencing outcomes.^22 57 86^ Powerful stakeholders, such as the Indian Medical Association, have opposed legislation affecting their commercial interests^53 54 66^; in contrast, some relatively qualified providers campaign for tighter regulation to constrain the operation of their less qualified counterparts.^22 53 60^ Politicians or officials with investments in the private sector may also undermine regulations.^54 80^

Despite these challenges, several enablers have been identified to strengthen regulatory outcomes. These include getting buy-in across multiple regulatory agencies, ensuring regulation is perceived as fair and transparent, digitising processes, strengthening logistics, and supporting and incentivising providers to comply.^22 41 60 65 85^ There is also interest in the potential of ‘decentered regulation’, involving multiple bodies such as accreditation agencies, insurers and even online marketplaces, though this can be disjointed.^54 57^ Finally, successful regulation requires detailed stakeholder analysis, alliance building and political mechanisms to prevent undue influence by lobby groups.^22 57^

#### Contracting and purchasing

Governments’ use of public funds to purchase or contract services from private providers has been justified by the need to expand coverage quality, efficiency and responsiveness.^4^ In the past 20 years, the growth of social health insurance programmes in LMICs has led to a substantial increase in public purchasing agencies contracting both private and public facilities to provide services for enrolled members.^87^ Other common forms have included contracting networks of FBO facilities in Africa,^88^ NGOs to manage primary care in crisis-affected settings,^4 38 89^ private facilities to provide specialised services such as dialysis,^4 52^ ‘management contracts’ for private organisations to provide services within government facilities^62 89^ and a surge in contracts for testing and treatment during the COVID-19 pandemic.^63^ Longer term ‘Private Financing Initiative’ contracts have also been used to leverage private funds for health facility construction or refurbishment.^449 90^,^92^ Contracting provides potentially powerful governance opportunities to influence private provider behaviour, but historically payments have often been based on past expenditure or norms, with little consideration of performance or regulatory compliance.^50 93 94^ In recent decades, a strong emphasis has emerged on ‘strategic purchasing’, which includes selecting facilities meeting specified quality standards, using payment methods to incentivise efficiency and quality, and enhanced performance monitoring.^2 48 50 87 93 95 96^

Evidence on the effectiveness of contracting compared with government provision in LMICs is mixed. A 2018 Cochrane review included only two studies: a 2015 controlled before-after study in Guatemala of contracting mobile clinic services to NGOs which found no impact on utilisation or service delivery, and a 2006 RCT in Cambodia of contracting district health services to INGOs, from before our review period.^30^ A more recent difference-in-difference study found contracting primary care services to NGOs in Brazil increased utilisation and reduced hospitalisation for preventable disease (Greve and Coelho 2017, cited in ref 97). Reviews with less restrictive inclusion criteria have generally identified evidence that contracting improved utilisation, input availability and patient satisfaction, but lacked evidence on clinical quality.^29 62 97^ However, Rao *et al* report mixed evidence on utilisation, finding that private providers faced many of the same service delivery challenges as their public sector counterparts.^89^

The key question for many governments may be how best to contract, rather than whether to contract, given that gaps in public provision and political realities may make contracting inevitable.^29 89^ There is little quantitative evidence on the relative effectiveness of alternative contracting mechanisms (an exception is the literature on the effectiveness of pay for performance^98 99^ but this primarily concerns public facilities). However, substantive insights are available from qualitative data and expert opinion, with several recent papers synthesising this evidence across multiple LMICs.^32 89 93 94^ They find that a first step to effective contracting is contract design, which should include appropriate and balanced incentives for facility enrolment, quality of care, efficiency and equity, plus referral and gatekeeping guidelines, and mechanisms for redress, all transparently published online.^32 87 93^ Care should be taken to reduce perverse incentives for overprovision or underprovision, unnecessary admissions and referrals, selecting out high-risk cases, and additional patient charges.^52 74 87^ However, in practice, reimbursement tariffs below cost and late disbursements were an important constraint on provider performance,^3264 89 93 100^,^103^ distorting provider incentives, increasing user charges and negatively affecting public–private relationships.^32 93 101 103^ In some countries, donors played an important role in addressing funding gaps, though this also risked sustainability challenges and low government ownership.^29 32 89 93^

Contract governance was often hampered by challenges in monitoring performance and applying sanctions.^29 78 89 90^ In some cases, third-party monitoring by NGOs or accreditation agencies has been used to strengthen oversight,^89 90 93^ and investment in digitised and automated processes has also been recommended to enhance monitoring and transparency.^32 104 105^ However, authors note the value of ‘relational contracts’ in some contexts, where specific contract stipulations are subordinated to building a trusting partnership, examples being contracts with African FBO facilities, though this may risk weak accountability.^88 89^

Fragmentation among purchasers was argued to be a key barrier to effective contracting, with contracting by multiple social and private insurance programmes and other schemes creating uncoordinated provider incentives and increasing administrative costs.^87 93 94^ Greater purchaser consolidation, or failing that, coordination, has been recommended to strengthen incentives and accountability.^93^ High fragmentation on the provider side (ie, a very high number of small facilities) can also increase the costs and complexity of enrolling facilities and monitoring contracts,^2 100 105^ with suggestions that greater provider organisation or consolidation would be beneficial, perhaps through intermediaries such as NGOs, HMOs or provider associations.^2 88 93 100 105 106^

Finally, it is argued that effective governance of purchasing systems requires a strong ‘task network’ of organisations, with clearly delineated responsibilities across purchasers, social health insurers, accreditation bodies, regulators and MoH departments.^94^ However, in practice, capacity was limited (see the Capacities for governance section), agency roles were often unclear and delays in development of quality standards and information systems hampered purchaser performance.^32 94 103^ The way in which purchasing agencies themselves are governed was also argued to be important.^32 93^ Although there was debate over the optimal degree of autonomy the purchaser should have from the MoH, there was consensus that the purchaser be subject to a clear accountability framework, specifying the strategic goals, governing laws and regulations, financial controls and transparency requirements.^32^ However, in practice, concern was expressed about political interference and the influence of vested interests in purchasing mechanisms (see the Foster relations section).^32 72 89^

### Foster relations

Foster relations covers the establishment of inclusive policy processes that involve a broad range of stakeholders including the private health sector.^15^

The literature covers a variety of approaches to including the private sector in policy processes. Some studies focus on donor-driven public–private dialogue platforms—often established to support the delivery of specific programmatic objectives.^45 48 107^ Others focus on policy processes related to specific reforms—for example, contracting arrangements,^51 78 93 101 104^ public–private partnerships for capital investments^42 44 108^ and regulatory change.^50 55 81^ In general, inclusion of the private sector in policy processes is considered to be a positive component of governance—generating benefits in terms of information exchange, trust building and balancing of interests.^8^ However, the literature also points to the threats posed by private sector influence activities in relation to governments’ policy goals,^8 59 72 81 109^ including in relation to equity of access and financial protection.^59^

It can be considered desirable for governments to engage with private sector associations, rather than individual private actors.^41^ However, the extent to which the insights, perspectives and interests of diverse stakeholders—and not only the most powerful—are represented by such entities can vary.^54 81^

A key message emphasised in parts of the literature is the importance of ensuring that policy processes are open, inclusive and transparent. Where public–private dialogue takes place ‘behind closed doors’, this is observed to create risks of state capture, bias and corruption.^42 72 90 101^ Non-transparent influence from powerful producer interests (eg, by the owners of investor-owned hospitals or private insurance companies) is observed to result in undue influence on policy making, to the potential detriment of UHC.^59^ To mitigate such risks, it has been argued that governments need to develop mechanisms for engaging with a broader range of stakeholders—including inter alia patients, social insurance recipients, civil society organisations (CSOs), etc, alongside private sector interests.^54^

### Build understanding

Build understanding concerns the government’s access to and use of data on the private sector.^15^

The literature records multiple efforts by governments and other stakeholders (notably donors) to improve governments’ access to data, alongside some of the challenges faced in doing so. Studies from multiple countries document a range of regulations that oblige private providers to collect and share data with state authorities.^41 45 56 81 104^ For example, facility licensing criteria may include a requirement for the private sector to provide data.^103^ This can include data on matters of public health importance, including reportable diseases such as HIV, malaria and TB; and public health programmes such as family planning and immunisation.^110^ In relation to service delivery more generally, access to data may be lacking even in service domains in which the private sector accounts for a significant proportion of provision (such as maternity care in Uttar Pradesh, India).^111^ The literature indicates that where information sharing is voluntary rather than mandatory, compliance can be limited^61 81 104 112^—though there is some evidence that the situation is improving, in part due to technological developments in DHIS2 modules or other health management information system (although reporting into DHIS2 often remains paper based).^8^ Financial incentives can encourage reporting—for example, if information provision is necessary for reimbursement under state purchasing arrangements.^59^

Data collection challenges may be driven by a number of factors, including a lack of trained personnel, high staff turnover rates, the burden of paper-based reporting (which remains common in LMICs), and uncertainty and/or misunderstanding about the purpose or value of reporting.^46 61 113^ Studies also point to the lack of interoperability between data systems as a key challenge.^114^ However, Gautham *et al* suggest that, while private facilities sometimes feared information disclosure, they were willing to share data if asked officially, if the process was simple, and if they were assured of confidentiality.^111^

### Align structures

Align structures focuses on government action to ensure alignment and coordination between the private and public sectors in service delivery.^15^ Approaches include engaging the private sector in quality of care initiatives, priority health programmes and referral networks. Use of and adherence to clinical and quality of care guidelines may be required for participation in national health insurance programmes,^93^ and licensing, as well as being incentivised through public training programmes, sometimes linked to programmes focusing on specific issues, such as combating antimicrobial resistance.^115^ Other examples include involvement in disease control,^2^ immunisation^116^ and family planning programmes,^47^ where private providers receive benefits such as training, equipment and supplies in exchange for compliance with notification and referral requirements, as well as meeting quality standards.^117^ There is also a growing literature on COVID-19 that looked at the contribution of the private sector during the pandemic, which is seen as one factor supporting effective responses in some settings.^63 84^

The literature provides limited evaluative evidence, especially on inclusion in referral networks. Private sector contributions to immunisation programmes vary, with mixed service quality reported in different countries.^118^ There is some evidence that private providers can fill gaps in service provision—for example, for immunisation in conflict-affected settings,^116^ and that they can offer advantages in reaching groups facing stigma, such as adolescents seeking reproductive care.^47^ However, quality criteria relating to general healthcare processes are noted to be commonly absent or not monitored, though some countries, such as South Africa, have introduced comprehensive quality criteria applicable to both public and private facilities.^22^ If there is no routine process of inspection or monitoring, the incentive to comply with evidence-based guidelines and referral regulations is limited.^59 81^

Enabling factors for ensuring effective alignment include mutual trust^63^; collaborative partnerships with clear roles and expectations; engagement of relevant professional and training bodies^115^; clear incentive structures favouring alignment for all actors; inclusion of the private sector in planning, supervision and reporting systems^116^; regular monitoring of quality metrics tied to provision of goods, such as vaccines^118^; and addressing barriers faced by private providers, including access to training and regulatory information.^47^ Alignment requires resourcing and leadership support,^115^ and deficits or delays in support and lack of recognition of the private sector’s contribution are demotivating.^119^

### Nurture trust

Nurture trust requires governments to safeguard the rights and financial welfare of the public through mechanisms to strengthen the public voice in health system governance, address patient complaints and provide legal redress, with many of these mechanisms covering both public and private providers.^15^

These mechanisms may be underpinned by patients’ rights charters or laws,^50 81 102^ such as the Indian Charter of Patients’ Rights.^54^ Opportunities for patient voice may occur through annual general meetings of social health insurance agencies, hospital boards, public consultations, feedback surveys and review apps.^2 62 71 93 95^ Complaint mechanisms include telephone hotlines and online portals,^57 94 120^ or ombuds offices,^2 81 102^ and patients or their advocates may also sue providers.^4 57 62^

Examples of well-functioning voice and compliant mechanisms were rare, with those identified often ad hoc, ineffective, distrusted or having low public awareness or participation.^50 56 75 94 102 121^ However, there were some positive exceptions. Indonesia’s LAPOR! (REPORT!) platform had reportedly been widely used to voice citizens’ views and submit complaints about health services,^65^ and Thailand’s National Health Assembly was said to provide a mechanism for the public to constrain private sector influence on policy.^71^ Thailand was also said to have robust systems for involving patient interest groups on particular diseases, and a well-functioning telephone helpline for social health insurance members,^94^ though the literature lacked information on why these had worked well.

An underlying barrier to effective public accountability is that patients often lack good information about quality of care, and on their rights and accountability mechanisms, with poorer and more vulnerable consumers particularly disadvantaged.^56 57 94^ It is argued that efforts to address this could include display of patient rights at facilities, facility scorecards and development of online mechanisms.^2 4 65 81^ CSOs, NGOs and patient groups may also facilitate interaction, and in some cases have official monitoring roles.^2 93^ However, there is considerable variation in how well different communities are represented, and capture of such mechanisms by local elites is possible.^57^

In some contexts, such as India and Thailand, consumer litigation had become a prominent regulatory tool, though fear of being sued was argued to lead to increasingly precautionary and ‘defensive’ medical practices, pushing up costs.^54 62 66^ Legal redress also requires citizens to have sufficient resources to pursue claims, and accessible court services, but this is not always the case in practice.^57^ For example, India’s district-level consumer courts were expected to facilitate quick and local resolution, but in practice the process could be lengthy and costly, with perceptions of a bias in favour of clinicians.^4 62 66^ In the absence of effective redress mechanisms, some disgruntled users were sharing grievances through social or press media, and, worryingly, violent attacks on healthcare workers had become a concern in some settings.^54 56^

### Capacities for governance

Across the governance behaviours, capacity to operationalise governance mechanisms was described as inadequate. In some areas, clear skills deficits were highlighted. For example, skills gaps for contracting included legal, clinical and financial risk management, claims data analysis, clinical coding and pricing, and performance monitoring and enforcement,^8 38 39 41 42 48 51 62 75 81 94 101 102 104 122^ while regulation and accreditation required skills in facility registration, inspection, and enforcement,^41 50 81 95 102 123^ and quality improvement/assurance.^42 48 95 123^ In addition to these technical skills, management and leadership skills were also noted as important enablers for governance, such as the ability to make good-quality decisions, innovate and manage change.^32 59^ However, challenges to skills development included high staff turnover, inadequate succession planning and loss of institutional memory.^81 102^

Inadequate capacity was also reported in the organisational processes and systems required for governance,^8 41 42 48 51 102 104 122^ meaning that, even where governance policies existed, most countries lacked capacity for implementation and enforcement.^41^ However, there was stronger capacity to govern the private sector within some vertical disease programmes, supported by external funding.^2 8^

Public actors reportedly faced particular challenges when operationalising governance in decentralised contexts. Local regulatory entities almost always reported insufficient human and financial resources for monitoring and enforcing regulation,^2 42 51 66 77 81 101 102 122^ and local actors were not involved in policy development, and sometimes did not clearly understand policy objectives.^2^ This may render local actors vulnerable to undue influence from local relationships, which are far more ‘present’, resulting in power imbalances during contracting and compliance (see Foster relations and Enable stakeholders sections).^48 51 81 82 122^ An evaluation of regulatory failures recommended the separation of the public health and regulatory functions at the local level, as they require distinct skill sets, and fundamentally different relationships with providers.^66 90^

Capacity constraints on the part of private providers also affect their ability to engage in governance mechanisms, particularly for small, individual or rural providers, who lack the resources for regulatory compliance.^8 43 46 48 51 90 123^ These providers report not receiving training on government reporting systems and processes, and lack the capacity to collect, maintain and share mandatory data with regulators.^2 46 48^ In some countries, donor-funded initiatives have worked with private providers to address these gaps.^43 45 46 107^ A lack of ‘collaborative capacity’ also emerged within and between the public and private sectors,^29 42 66 81 101^ with the observation that cultural, relational and institutional ‘software’ are crucial for legitimacy, feasibility and enforceability of governance mechanisms.^85^ Effective governance was reported as requiring trust, cooperation and collaboration capacity, both across the sectors and between agencies, networks, civil society and communities.^23 65^

Efforts to enhance governance capacity generally involved donor-supported technical assistance programmes providing human resources, operational processes, training, and stakeholder forums and engagement,^43 45 46 107^ sometimes including the establishment of specific public–private partnership units.^8 43 44 46 48 108 124^ While improvements in governance were reported, concerns were raised about sustaining this engagement at scale when external funding ceased,^43 46^ and the degree of public ownership in governance strategies that have had strong external input.^4 22 57^

## Discussion and conclusion

This review synthesises the literature relevant to the governance of the private healthcare sector in LMICs, organised according to WHO’s six governance behaviours. The findings have informed the development of a ‘Progression Pathway for the Governance of Mixed Health Systems’, which aims to enable countries to assess their governance arrangements, prioritise future actions and track progress.^109^

A strength of the review was the inclusion of a wide range of literature in terms of study design, data collection methods and publication type, reflecting the valuable evidence that can be identified on this topic from not only rigorous research studies, but also from descriptions of policy and practice, and reflections of actors directly engaged in governance. Given this variability in methodological approach, we chose not to base inclusion on a formal quality assessment which could have led to exclusion of certain forms of valid evidence. We also took a broad approach to the definition of ‘governance’, guided by the WHO governance behaviours. The nature of the topic precluded a tightly targeted search strategy, with our initial searches identifying over 11 000 articles. We therefore used machine learning technology to order the screening of the papers, in practice screening about a fifth of the search results. It is possible that relevant articles were not screened before our stopping rule was reached. However, we supplemented database searches with articles identified by experts on the literature, leading to some degree of confidence that the most significant articles were included. The breadth of the governance topic also presented some challenges at the synthesis stage, particularly for the enable stakeholders literature on regulation and contracting, as these are two extremely broad areas of literature in themselves. To maintain feasibility, we drew where possible on existing literature reviews or evidence syntheses on these topics, while also including individual empirical papers to elaborate key issues.

The literature had considerable geographical breadth, covering a high number of LMICs, though with very little on fragile and conflict-affected settings. There was also breadth in terms of governance topic, though some neglected areas included private health insurance regulation, taxation, public accountability mechanisms and governance of recent market developments, such as digital health, medical tourism, hospital and pharmacy chains and private equity investors.^57 125 126^

The literature drew primarily on qualitative methods and document review, typically including interviews with high-level stakeholders on their perceptions, with few in-depth qualitative studies of the operation of governance mechanisms on the ground.^54 85 127^ Data on stakeholder perceptions may be subject to social desirability bias with, for example, government staff wanting to be seen as effective regulators, and private providers having strategic interests in arguing for less regulation. In a number of cases, government staff and their advisors were authors, which could be a further source of bias. However, this literature did include extensive coverage of perceived problems with governance approaches. There was limited detail on the intensity of implementation of governance mechanisms (eg, number of meetings, frequency of inspections, sanctions implemented, etc), and only one rigorous costing study.^69^ There was a particular lack of quantitative evidence on the impact on governance outcomes (no quantitative measures of governance were presented), provider behaviour or UHC indicators, with only a couple of exceptions.^68 70^ This may reflect the challenges of studying governance; it is difficult to measure, and legal changes or health system reforms cannot be easily piloted or withheld from control groups. In sum, care is needed in interpreting the literature to identify credible evidence of ‘what works well’, as opposed to the claims of those involved in implementation or the many opinions on offer of what could be improved. Given the patchy nature and varied quality of the evidence base, identifying firm conclusions about patterns of findings across geographical regions or income levels is challenging. Future research should include more in-depth qualitative explorations of governance mechanisms and more rigorous documentation of context, implementation and outcomes.

A number of important lessons emerged from the review. First, effective performance of each governance behaviour requires a clear vision for the private sector’s role in the delivery of health system goals, together with specific mechanisms to enable this. Second, synergies can be exploited between governance mechanisms, such as linking contracting with regulatory compliance, or including data submission as a licensing requirement. Inadequate capacity, at the individual and organisation levels, was one of the most prominent themes, reflecting a persistent failure to see private sector governance as core to the government’s role. The skills required are well documented, but greater evidence is needed on how these can be developed, especially in devolved contexts and without donor support. Greater emphasis is also needed on developing strong (and ideally independent) institutions for regulation, purchasing and quality assurance. Another clear lesson is the importance of inclusive and transparent policy processes. Public–private dialogue ‘behind closed doors’ creates risks of state capture, bias and corruption. To balance legitimate stakeholder interests, the focus should be on purposeful deliberation in multistakeholder platforms, including patients, social insurance recipients and CSOs. The public sector also needs to be held accountable in its governance actions, including adherence to contract terms, transparency in tender and regulatory practices, and control of favouritism and other corrupt practices, an area which merits much greater innovation.^128 129^ A final lesson is that governance choices shape not just current private sector behaviour, but also the future development of the health system. Once a large and powerful constituency of private facilities or health insurers has developed, it can be particularly challenging to make progress towards UHC. Conversely, sustained effective governance has the potential to shape market development in line with health system goals, with the literature highlighting the many priority areas where further action is required to achieve this outcome.

## Supplementary material

10.1136/bmjgh-2024-015771 online supplemental file 1

10.1136/bmjgh-2024-015771 online supplemental file 2

## Data Availability

All data relevant to the study are included in the article or uploaded as supplementary information.
