# Supplementary material for: Approaches, enablers and barriers to govern the private sector in health in low- and middle-income countries: a scoping review
Source: BMJ Glob Health. 2024 Nov 13;8(Suppl 5):e015771. doi: 10.1136/bmjgh-2024-015771 (PMC11599734; doi:10.1136/bmjgh-2024-015771)
Supplement: online supplemental file 2 [file bmjgh-8-Suppl_5-s002.docx]

# Title: Approaches, enablers and barriers to govern the private sector in health in low- and middle-income countries: a scoping review

Supplementary material

Table 1: Inclusion/ exclusion criteria

|  | Inclusion criteria | Exclusion criteria |
| --- | --- | --- |
| Private healthcare sector | Papers should concern the private healthcare sector, defined as follows:   - Engaged in the delivery or finance of health service-related goods and services (e.g. health facilities, pharmacies, drug shops, telehealth providers, health insurance firms etc). - Can be formal or informal, qualified providers, and for-profit or not-for-profit. | - Manufacturing sector for pharmaceuticals, medical devices, and other commodities. - Provision of unhealthy commodities (e.g. sugary drinks, tobacco). - Health promotion activities that go beyond the health sector (e.g. water and sewerage, clean air, green spaces). - Social care (e.g. long-term residential care for the elderly who need living support rather than healthcare). - Training institutions for healthcare workers. |
| Governance | - Relate to one or more of the WHO Governance Behaviours (e.g., Deliver strategy, Build Understanding, Enable Stakeholders, Foster Relations, Align Structures, and Nurture Trust) - Examine governance at national or sub-national (e.g. state or province) level. | - Papers that describe the private sector in terms of numbers, utilisation, quality, cost, etc. - Governance of multinational private sector engagement/partnerships (e.g. Gavi). - Development impact bonds and other similar financing mechanisms. |
| Countries | LMICs from all WHO regions. |  |
| Study type | - Literature reviews (systematic and otherwise). - Papers that draw on/synthesise a body of empirical experience. - Empirical studies of any kind or study design, including both descriptive studies and evaluations of governance mechanisms, using qualitative and/or quantitative data, and any outcome measure. | - Commentaries and opinion pieces, unless considered critical sources of information on enablers or barriers to effective governance of the private sector. |
| Publication status | - Peer-reviewed articles. - Books. - Grey literature (e.g. policy papers, reports). |  |
| Date of publication | - 1 January 2010 to 27 January 2023. - Selected earlier studies with substantial significance for the evidence base, as assessed by relevance and frequent citation. |  |
| Language | - All languages. |  |

**Search strategy for published articles**

- 1. **Medline Ovid**

| **S. No.** | **Theme** | **Search terms** |
| --- | --- | --- |
| **1.** | Private sector | private healthcare OR private health OR Private sector OR informal sector OR for-profit OR not-for-profit OR public-private OR faith-based OR non-governmental organisation OR retail OR charity OR private organisation* OR profit-driven OR privatisation OR private provider OR private health insurance OR private medical insurance OR private hospital* OR private clinic* OR private pharmac* OR drug shop* OR drug seller* |
| **2.** | Limit | 2010- Present |
| **3.** | Governance | Governance OR stewardship OR regulat* OR engagement OR oversight |
| **4.** | Limit | 2010- Present |
| **5.** | MeSH terms | Public–private sector partnerships/ |
| **6.** | Limit | 2010- Present |
| **7.** | MeSH terms | Private sector/ |
| **8.** | Limit | 2010- Present |
|  | (2 OR 6 OR 8) AND 4 | Private sector (including MeSH terms) and governance |

- 1. **Scopus**

| **S. No.** | **Theme** | **Search terms** |
| --- | --- | --- |
| **1.** | Private sector | ( TITLE-ABS ( {private healthcare} OR {private health} OR {private sector} OR {informal sector} OR {for-profit} OR {for profit} OR {not-for-profit} OR {not for profit} OR {not for-profit} OR {public-private} OR {public private} OR {faith-based} OR {faith based} OR {non-governmental organisation} OR {non-governmental organisations} OR retail OR charity OR {private organisation} OR {private organisations} OR {profit-driven} OR {profit driven} OR privatisation OR {private provider} OR {private providers} OR {private health insurance} OR {private medical insurance} OR {private hospital} OR {private hospitals} OR {private clinic} OR {private clinics} OR {private pharmacy} OR {private pharmacies} OR {drug shop} OR {drug shops} OR {drug seller} OR {drug sellers} ) ) |
| **2.** | Governance | ( TITLE-ABS ( governance OR stewardship OR regulat* OR engagement OR oversight ) ) |
| **3.** | Health specific | ( TITLE-ABS ( health OR medical ) ) |
| **4.** | Limit | PUBYEAR > 2009 AND PUBYEAR < 2023 |
| **5.** | (1 AND 2 AND 3) AND 4 | Private sector and health and governance |

- 1. **Web of Science**

| **S. No.** | **Theme** | **Search terms** |
| --- | --- | --- |
| **1.** | Private sector | (TI= (“private health*” OR “Private sector” OR “informal sector” OR “for-profit” OR “for profit” OR “not-for-profit” OR “not for profit” OR “not for-profit” OR “public-private” OR “public private” OR “faith-based” OR “faith based” OR “non-governmental organisation*” OR retail OR charity OR “private organisation*” OR “profit-driven” OR “profit driven” OR privatisation OR “private provider*” OR “private health insurance” OR “private medical insurance” OR “private hospital*” OR “private clinic*” OR “private pharmac*” OR “drug shop*” OR “drug seller*” )) OR (AB=(“Private sector” OR “informal sector” OR “for-profit” OR “for profit” OR “not-for-profit” OR “not for profit” OR “not for-profit” OR “public-private” OR “public private” OR “faith-based” OR “faith based” OR “non-governmental organisation*” OR retail OR charity OR “private organisation*” OR “profit-driven” OR “profit driven” OR privatisation OR “private provider*” OR “private health insurance” OR “private medical insurance” OR “private hospital*” OR “private clinic*” OR “private pharmac*” OR “drug shop*” OR “drug seller*” )) |
| **2.** | Governance | (TI=(Governance OR stewardship OR regulat* OR engagement OR oversight)) OR (AB=(Governance OR stewardship OR regulat* OR engagement OR oversight)) |
| **3.** | Health specific | (TI= (health or medical)) or (AB= (health or medical)) |
| **4.** | Limit | Manually install in 2010-2023 limit |
| **5.** | (1 AND 2 AND 3) AND 4 | Private sector and health and governance |

**Organizational repositories searched for grey literature**

Results for Development, WHO, World Bank e-Library, Institute of Development Studies, University of Sydney, Lee Kuan Yew School of Public Policy
